# Research on the Effect of Executive Incentive Institutional Innovation on the Cost of Equity—Evidence From Chinese Listed Companies

**DOI:** 10.3389/fpsyg.2021.686955

**Published:** 2021-06-08

**Authors:** Jin Wang, Jie Deng

**Affiliations:** ^1^College of Finance and Economics, Guangdong Polytechnic Normal University, Guangzhou, China; ^2^Department of Finance, Guangdong University of Foreign Studies, Guangzhou, China

**Keywords:** institutional innovation, academic education, cost of equity, entrepreneurial spirit, innovative ideas

## Abstract

Executive incentive has long been a hot topic among academics and practitioners. With the continuous development of China's manager market, the spirit of innovation and entrepreneurship among executives has exerted a greater influence on corporate performance. Enterprise innovation is an important part of the entrepreneurial spirit. Moreover, China's supply-side reforms and compensation system of the state-owned enterprises (SOEs) have been advanced and innovative. Therefore, based on the manager human capital theory and the organizational innovation theory, and using 15,492 firm-year observations from China's Shanghai and Shenzhen A-share listed companies for the period 2005–2018, we constructed various models, including the Gorden model, the Growth Rate of Price–Earnings Ratio model (PEG), the Ohlson and Juettner-Nauroth model (OJ), and the Capital Asset Pricing model (CAPM), to measure the cost of equity. We investigated the effect of the institutional innovation of executive incentives on the cost of equity, and the heterogeneous influence of China's special property rights system on the relationship between the two. We found that the innovations of the executive incentive system have a positive governance effect on the cost of equity. In particular, executive compensation incentives significantly reduce a company's equity costs. We also find that the state-owned property rights may weaken the positive effect of institutional innovation of executive incentives. Furthermore, China's executive incentives system and corporate governance mechanism are imperfect; and therefore, institutional innovation is a matter of great urgency and more innovative ideas for the manager market need to be introduced. China's listed companies should give full play to the spirit of innovation and entrepreneurship, constantly innovating incentive-based compensation systems of companies, and establishing a scientific and innovative concept of the cost of equity. The findings are robust after controlling for potential endogeneity concerns.

## JEL Classifications

G15; G30; G34

## Introduction

For China in the economic transition, the key to overcoming the middle-income trap is to enhance the innovation ability and productivity of the Chinese firms, promoting intensive margin growth. There is an urgent need to innovate executive incentive systems in Chinese enterprises, especially among the SOEs. In 2013, the third plenary session of the 18th Central Committee of China emphasized the importance of innovating modern compensation systems for Chinese SOEs. According to a 2013 survey, the average salary of executives in the financial industry was 90 times that of common citizens, and even the daily salary of a top executive equaled the 3-month salary of a top teller. In 2014, Xinhua reported that the main company leaders earned salaries about 12 times that of other employees in central enterprises. Thus, a plan to reform the compensation systems of centrally administered enterprises was introduced in January 2015. The first batch of institutional innovation involved 72 SOEs, including 53 SOEs, such as Petrochina, Sinopec, and China mobile, and 19 other financial and railway enterprises. During the same year, Kweichow Moutai, a company in the liquor industry, appointed 11 vice presidents, and the annual salaries of 14 liquor executives were significantly higher than those of the executives in other enterprises in the same industry. Such news about unreasonable executive incentives is bound to become even more common in China. It has aroused doubts and discussions within the public and among academic researchers. Thus, we should consider, what is the purpose of executive incentives?

Since Jensen and Murphy (1990) first studied executive incentives, an increasing number of scholars have focused on this topic (Dyreng et al., 2010; Bebchuk et al., 2011; Adhikari et al., 2015; Chen et al., 2018; Fang et al., 2018). However, we should pay more attention to the goal of the incentive mechanism, that is, the coordination of interests between shareholders, management, employees, and creditors (La Porta et al., 1997; Shleifer and Vishny, 1997; Bertay and Uras, 2020; Castro et al., 2020). Capital cost is one of the key factors in management's financial decision making (Collins and Huang, 2011; Hasan et al., 2015; Shen and Zhang, 2020; Luong et al., 2021). It also affects investors' resource allocation decisions (Leone et al., 2006; Pástor et al., 2008), restricting the improvement of corporate production efficiency.

Meanwhile, the specific plan for China's “supply-side” structural reform in 2016 emphasized that the substantive goal was to reduce the capital cost and tax burden of the firms to ultimately improve social productivity. However, due to historical reasons, most of the Chinese companies have not set up a scientific concept of capital cost. This directly restricts the comprehensive competitiveness among the Chinese firms. Capital cost not only affects investors' interests, but also the basic criterion for drafting all kinds of financial policies, such as investment and dividend policies, so it is important for firms to establish a scientific and innovative concept of capital cost. Chinese firms can optimize corporate governance and productivity levels after clarifying the scientific process of “manager's behavior—capital cost—investor wealth—creating firm value.”

Research on executive incentives and capital cost has mainly explored the effects of corporate performance on executive incentive systems (Chen et al., 2018; Fang et al., 2018; Abudy et al., 2020; Chowdhury et al., 2020; Carter et al., 2021; Gao et al., 2021; Ren et al., 2021). For example, Chen et al. (2018) investigated the effect of corporate financial distress risk on the initial compensation contracts of new executives. Fang et al. (2018) verified the effects of major shareholder ownership on executives' excess compensation. With respect to capital cost, recent studies have focused more on the relationship between these costs and information disclosure, CEOs' internal debt, and the social relations between executives and directors (Collins and Huang, 2011; Hasan et al., 2015; Shen and Zhang, 2020; Luong et al., 2021). They often have ignored the impact of executive incentive systems on corporate equity cost.

Against the background of China's supply-side reforms, we investigated the relationship between executive-incentive-system innovation and the cost of equity, as well as the effects of China's special property-rights system on this relationship. Our conclusion provides a theoretic guidance for an in-depth understanding of the capital cost and offers empirical evidence for the necessity and the feasibility of the innovation of executive incentive systems among Chinese firms. We also offer a new explanation of the importance of entrepreneurship in corporate performance. Entrepreneurship is the inner impulse of business people to pursue innovation and indicates that entrepreneurs are willing to meet market challenges, break market equilibrium, and discover new opportunities in changes to policy and the business environment. Entrepreneurship has a significant impact on corporate behavior and financial decisions, promoting the differentiation of enterprises (Wu and Hsu, 2018; He et al., 2019; Howell, 2019). We show that the innovation of executive incentive systems is conducive to creating a favorable environment for institutional innovation, which helps give full play to entrepreneurship, further reducing corporate costs and improving corporate performance.

This study contributes to several strands of research. First, our research further explores the relationship between executive incentives and equity cost. Existing studies have focused on the impact of the characteristics of a firm, such as political association, information disclosure, life cycle, CEOs' internal debt, and the social relations between executives and directors on equity costs (Boubakri et al., 2012; Hasan et al., 2015; Ng and Rezaee, 2015; Gupta et al., 2018; Shen and Zhang, 2020; Luong et al., 2021); however, work that directly explores the relationship between executive incentives and equity cost is rare. For example, Luong et al. (2021) used quasi-natural experiments to study the impact of social relations among executives, directors, and securities traders on the cost of equity. Shen and Zhang (2020) investigated the relationship between CEOs' internal debt and the cost of equity. Ahmed et al. (2018) found that the investment of corporate derivative instruments can reduce the cost of equity. Thus, this paper is the first to fully discuss the governance effect of executive incentives (mainly executive compensation incentives) on the cost of equity, considering China's unique characteristics.

Second, this paper further investigates the effects of property rights on the relationship between executive incentives and equity cost. Corporate property rights in China are different from those of developed countries. They greatly affect the capacity and financial resources of the Chinese firms. Serious “credit discrimination” exists in China's financial market; in other words, banks favor SOEs while discriminating against private enterprises when lending. This is reflected in two aspects: SOEs have relatively easy access to loans, and loan costs for SOEs are lower. Thus, property rights can be regarded as one of the unique characteristics of Chinese enterprises. Furthermore, our data show that the amount of research and development (R&D) investment and the number of patent applications among SOEs is significantly lower than for non-SOEs, indicating that China has not yet established an effective innovation incentive mechanism for SOEs. As a result, this paper has great practical significance for designing an innovation-oriented incentive system for senior executives of SOEs.

Third, this paper discusses the influence of executive traits or entrepreneurship on the cost of equity in China. It extends the research on corporate innovation, providing enlightenment on the entrepreneurial policies of emerging economies. The innovation of executive incentive systems can realize executives' self-value, promoting a virtuous circle between high-quality business startups and excellent entrepreneurial spirit as the core force in corporate institutional innovations. Therefore, executive incentive system innovation can more effectively stimulate the entrepreneurial spirit, improving the level of corporate innovation.

Fourth, our findings provide a reference for establishing an effective and innovative executive incentive mechanism. China's relevant literature has focused on the influence of executive equity incentives on corporate equity cost, and most studies have concluded that equity incentives aggravate agency costs. However, most executives hold few or even no shares in Chinese companies, as shown in our data, and the average rate of executives' shareholdings is only 5%. This indicates that the effectiveness of equity incentives in China needs to be further verified. Therefore, we directly investigated the impact of executive monetary compensation incentives on the equity cost, providing significant guidance for the innovation of executive incentive systems among Chinese SOEs.

This paper has several research limitations and suggests directions for future research. First, due to the low shareholding ratio of senior executives in Chinese enterprises, the effectiveness of equity incentives in China has not been fully verified. Therefore, this study reviewed the impact of monetary compensation incentives on the cost of equity. Other indicators, such as equity incentives, executive promotion incentives, and executive psychological incentives, can be used as indicators of executive incentive systems in future research (Chesney et al., 2020; Wruck and Wu, 2021). Second, the estimation of the cost of equity has always been a difficult and controversial task; as such, many estimation models have been proposed in the literature. Future studies can select the most effective model to further explore the influence of other executive traits on the cost of equity. Finally, future studies can pay more attention to the different effects on special enterprises, such as startups and high-tech companies, and the relationship between executive traits and the cost of equity.

The paper is structured as follows. Section Research Hypothesis develops the research hypothesis, and Section Research Design details the research design. Section Empirical Results presents the empirical results, and additional robustness tests are described in section Robustness Tests. Finally, section Discussion concludes the study.

## Research Hypothesis

### The Effect of Institutional Innovation of Executive Incentives on the Cost of Equity

The classical organizational innovation theory notes that entrepreneurs play a dynamic role in whether an innovation results in the success or failure of a company. The ability and motivation of managers performing strategic control are the keys to enterprise innovation (Hirshleifer et al., 2012; Aghion et al., 2013; Kong et al., 2020). Berle and Means (1932) were the first to suggest the separation of “ownership” and “management rights” among modern enterprises. They proposed the classic “agent–agent theory,” which stated that the “two rights” separation leads to information asymmetry between the principal and the agent, resulting in agency problems between shareholders and managers and higher agency costs. However, their theory has only been tested using a utility function rather than empirical research (Chen et al., 2018; Fang et al., 2018; Carter et al., 2021).

The groundbreaking empirical research on the agency theory is the contract between the principal and the agent as proposed by Jensen and Meckling (1976). This posits that there is a contractual relationship between the principal and the agent. The shareholder, as the principal, wishes to earn wealth by giving the manager a certain salary and remuneration, whereas the manager, as the trustee, wants to obtain the reward to realize their self-worth. However, during the implementation of the contract, the manager is likely to pursue the self-benefit and damage the interests of shareholders, resulting in the challenges of “moral hazard” and “adverse selection” (Jensen and Meckling, 1976; Biener et al., 2018; Borochin and Knopf, 2021). Thus, shareholders may reduce the investment risks by improving managerial incentives or strengthening supervision (Shen and Zhang, 2020; Luong et al., 2021).

As for corporate capital cost, Skaife et al. (2004) demonstrated that corporate governance factors, such as financial information transparency, audit committee independence, and board independence reduce equity cost, whereas other factors, such as equity concentration and the number of big shareholders increase equity cost. Chen et al. (2003) found that there is a significant negative correlation between information disclosure and equity financing costs as evidenced by Asian emerging capital markets. Chen et al. (2009) suggested that stronger shareholder rights systems, more effective boards of directors, and a better quality of financial information disclosure tend to reduce equity financing costs. Luong et al. (2021) found that the social relationship among executives, directors, and securities brokers reduces the cost of equity, and the influence is more significant in companies where executives hold a high amount of equity. Shen and Zhang (2020) identified a negative correlation between CEOs' internal debts and the cost of equity, and this relationship is more significant in companies with a high leverage ratio.

Corporate governance should guarantee the interests of investors and satisfy their remuneration (Shleifer and Vishny, 1997; La Porta et al., 2000; Denis and McConnell, 2003). Under the separation of two rights, information asymmetry exists between the management and the fund providers, such as shareholders and creditors (La Porta et al., 2000; Denis and McConnell, 2003; Shen and Zhang, 2020; Luong et al., 2021). Investors cannot distinguish the actual risk of their investment; thus, they seek a capital price protection mechanism to reduce their investment risk and guarantee investment interests. According to the risk level, investors require a certain return rate, producing corporate equity cost. This is the basic requirement for equity investors to grant their rights to use the fund, and it leads to an increase in the firm's financing costs (Kabir et al., 2013). Therefore, enterprises can motivate and constrain the management through various governance mechanisms.

Executive incentive is one of the most effective mechanisms to mitigate the agency problem (Graham et al., 2012; Chen et al., 2018; Page, 2018; Gilje et al., 2020). In the modern corporate governance mechanism, the most common goals of executive compensation incentives are as follows: (1) to ensure the interests of capital suppliers and maximize the wealth of shareholders (Shleifer and Vishny, 1997), and (2) to maximize the overall value of the company under the condition of achieving the first goal (Page, 2018; Abudy et al., 2020). In view of these goals, firms tend to incentivize and constrain top executives using executive incentive contracts. Specifically, the firms set a higher level of compensation to reduce the agency problem between executives and shareholders, encouraging the alignment of executives and shareholders' interests (Chen et al., 2013, 2018) and, eventually, a reduction in the cost of equity (Mishra, 2014).

Moreover, with the continuous development of the manager market in China, professional managers' reputations also encourage managers to work hard; that is, executives are most likely to work hard when they pursue self-interest maximization and self-worth realization (Murphy and Zabojnik, 2004; Chen et al., 2018; Shen and Zhang, 2020). The implementation of an effective executive compensation contract not only helps firms attract excellent talent, but also promotes the sharing of benefits and risks between the management and shareholders, mitigates the agency problem, and thus reduces corporate equity cost (Chen et al., 2013; Luong et al., 2021).

As mentioned, the level of executive incentive reflects the agency conflict between shareholders and the management to a certain extent. A higher executive pay may relieve these agency conflicts (Frydman, 2007; Gupta et al., 2018; Rjiba et al., 2021). Also, a firm with a lower equity cost may have retained more earnings to invest in better projects, increasing firm value and shareholders' wealth, and thus improving executives' rewards (Shen et al., 2010; James, 2013; Mishra, 2014; Chen et al., 2018; Gan et al., 2020).

Therefore, in this review, the authors emphasize that the executive incentive system, cost of equity, and the maximization of corporate value should be considered together. The more appropriate the executive incentive system, the higher the executive compensation level will be, and the lower the cost of equity. Based on the above analysis, the authors propose the following hypothesis:

*Hypothesis 1: Executive incentive system has a negative effect on the cost of equity*.

### The Effect of Property Rights on the Relationship Between Executive Incentive and Equity Cost

Most of the executives of Chinese state-owned firms are political officials. The compensation system for state-owned firms has always been controlled by the government—the only “regulating hand.” Salaries of executives of the state-owned firms are controlled by the government, so their compensation has a certain degree of stability, or salary stickiness (Gaver and Gaver, 1998; Jackson et al., 2008; Hoi et al., 2019). Relative to private firms, state-owned firms tend to pay more attention to social benefits, such as employment and environmental protection. In state-owned firms, management goals are more related to political tasks rather than firm value maximization.

Many studies have shown that the performance of private firms is better than that of the state-owned firms. In terms of external financing, private firms are more systematically discriminated against in terms of whether they refinance from the stock market or receive loan financing from banks (Kabir et al., 2013). Chen et al. (2015) also showed that there are significant differences in the effects of Extensible Business Reporting Language on the equity cost of SOEs and non-SOEs.

Given a company's cash flow, a decrease in equity cost means that the company is more likely to improve executive pay with an increase of firm value (Chen et al., 2013). Owner vacancy and the salary restriction system may weaken the negative relationship between executive compensation and equity cost in state-owned firms. Boubakri et al. (2012) stated that the cost of equity in politically affiliated companies is lower than in non-affiliated companies. Szarzec et al. (2021) discussed the impact of SOEs on economic growth in 30 European countries. They find that, when the national economic system is weak, the influence of SOEs on the economy is more harmful. Guan et al. (2021) found that the chairmen of SOEs have a significant impact on financial performance, and mixed ownership improves the efficiency of SOEs. Therefore, we expect that SOEs pay little attention to equity cost and have little incentive to reduce equity cost by increasing executive compensation.

Among private firms, executive incentive systems are more aligned with the development of the market. Private firms tend to suffer more competitive and greater external financing constraints. Thus, they are more willing to raise the level of executive pay to motivate the executives, reducing net payoffs and speculation. Additionally, private firms have stronger motivation to encourage executives to guarantee the interests of shareholders, ultimately mitigating the information asymmetry between the shareholders and the management, and thus reducing equity financing costs. Therefore, we propose the following hypothesis:

*Hypothesis 2: Property rights of state-owned firms weaken the effects of executive incentive systems on the cost of equity*.

## Research Design

### Sample Selection and Data Sources

We utilized corporate financial data from the China Stock Market & Accounting Research database (CSMAR), the analysts' earnings forecast data from the Wind database, and risk-free returns data manually calculated by Chinese banks' 1-year regular deposits, which are collected from the website of the People's Bank of China.

This study adopted four models to calculate equity cost: (1) the Gorden model (Gorden and Gorden, 1997); (2) the Growth Rate of Price-Earnings Ratio model (PEG; Easton, 2004); (3) the Capital Asset Pricing model (CAPM; Sharpe, 1964); and (4) the Ohlson and Juettner-Nauroth model (OJ; Ohlson and Juettner-Nauroth, 2005). The Gorden and PEG models are used in baseline regressions, whereas the CAPM and OJ models are used in robustness tests. Since China's analyst data used in the PEG and OJ models have been available since 2005, we use data for A-share listed companies listed on the Shanghai and Shenzhen stock markets from 2005 to 2018.

We processed the dataset as follows. First, in the financial industry, financial data, financial statements, and salary disclosures of the firms are very different from those of listed companies in other industries in China, so we excluded financial and insurance companies based on the Industry Classification Benchmark code. Second, China's main stock market is the Shanghai and Shenzhen A-shares (the stocks listed in China and issued in RMB), so we excluded B shares and H shares (non-RMB stocks issued in foreign countries or Hong Kong) with different characteristics. Third, we excluded companies with a null value for the main variables. Fourth, we winsorized corporate financial continuous variables at the 1% level after considering the influence of extreme values. Fifth, we excluded companies issuing shares for <2 years because the financial disclosure data of those companies were discontinuous. Finally, we excluded companies with an asset–liability ratio higher than one after considering high financial risks caused by excessive liabilities.

We also adopt the following additional methods to deal with the data:

1) In the Gorden model, we excluded data without an initial closing price, *P0*, and sustainable growth rate, *G*, and we excluded data with a negative sustainable growth rate and a sustainable growth rate greater than one.
2) In the PEG model, we excluded data without earnings per share *EPS*_1_, *EPS*_2_, and *P0*.
3) In the OJ model, we excluded data without a predicted EPS and *P0*. If *EPS*_1_ greater than *EPS*_2_ was predicted, we made *EPS*_1_ equal to *EPS*_2_, following Wang et al. (2014). If a negative value appeared under the square root, the equity cost was made to be equal to A to avoid missing values.

Following this, our final sample comprised 15,492 firm-year observations.

### Variable Setting

#### Executive Compensation Index

We used two indicators to measure executive compensation: (1) *pay*, the natural logarithm of the total salary of the top three executives; (2) *CEOpay*, the natural logarithm of the total annual salary of the general manager and CEO; and (3) *MPAY2*, the median of the total annual salary of the general manager in industry *i* at year *t*.

#### Equity Cost Index

The cost of equity was measured using two methods. The first is the basic model of financial management principles, namely, the “ex ante cost,” as used in the PEG, OJ, and Gebhardt, Lee, and Swaminathan (GLS) models (Chu et al., 2020; Pfister et al., 2020; Husser and Paulet, 2021). This kind of model is based on the idea that the value of a company's future dividends or cash flow discount is equal to the internal return rate of its stock price (Gebhardt et al., 2001; Hou et al., 2012). The second is the risk compensation method, or “ex post,” as used in the Gorden and CAPM models. Equity cost was calculated using historical data in the Fama–French three-factor model (Fama and French, 1993; Chen et al., 2013; Ahmed et al., 2018; Shen and Zhang, 2020; Luong et al., 2021; Rjiba et al., 2021). All five models provide a reasonable estimate of the cost of equity. However, the GLS model assumes that the ROE tends to equal the average ROE of the industry in a linear and equi-differential way, and the residual return remains unchanged after the twelfth period. Also, the calculation method using the higher-order equation is relatively complex. Thus, based on the method used by most scholars (Chen et al., 2013; Ahmed et al., 2018; Chu et al., 2020; Pfister et al., 2020; Shen and Zhang, 2020; Husser and Paulet, 2021; Luong et al., 2021; Rjiba et al., 2021), after considering the available parameters, we adopted four models—Gorden, PEG, CAPM, and OJ—to calculate the cost of equity as follows:

(1) Gorden model:

(1) $$\begin{array}{l} \text{r}{\text{e}}_{\text{t}}=\frac{\text{D}1}{\text{P}0}\;+\text{ G} \end{array}$$

where dividend payments and future sustainable growth determine a company's equity cost. *D*_1_ is the first year's dividend, and *G* is the growth rate of pre-tax dividends per share.

(2) PEG model:

(2) $$\begin{array}{l} {\text{R}}_{\text{t}}=\sqrt{\frac{\text{EPS2 }-\;\text{EPS1}}{\text{P0}}} \end{array}$$

Proposed by Easton (2004), this model estimates a company's hidden equity cost based on the company's future earnings forecast data. *EPS*_1_ and *EPS*_2_ are the company's earnings forecasts per share in the next one and 2 years. We select the latest earnings forecast data in each year—that is, December earnings forecasts—and average the forecast data of multiple analysts according to the same stock symbol to obtain the final earnings forecast. *P0* is the latest closing price at the end of the year.

(3) CAPM model:

(3) $$\begin{array}{l} \text{gqc}{\text{b}}_{\text{t}}=\text{R}{\text{f}}_{\text{t}}\;+\;{\text{β}}_{\text{t}}\;\times \;\left(\text{R}{\text{m}}_{\text{t}}-\text{R}{\text{f}}_{\text{t}}\right) \end{array}$$

where *Rf* is the annual risk-free return rate, β is the market risk calculated by the market's comprehensive annual β, and *Rm* is the market's annual return rate calculated by the market's comprehensive annual return rate and considering the reinvestment of cash dividends.

(4) OJ model:

(4–1) $$\begin{array}{l} \text{R}{\text{e}}_{\text{t}}=\text{A}+\sqrt{{\text{A}}^{2}+\frac{\text{EP}{\text{S}}_{1}}{\text{P}0}\left[\frac{\text{EP}{\text{S}}_{2}-\text{EP}{\text{S}}_{1}}{\text{EP}{\text{S}}_{1}}-\left(\text{γ}-1\right)\right]} \end{array}$$

(4–2) $$\begin{array}{l} \text{A}=\frac{\text{ γ }-\;1\;+\;\frac{\text{D1}}{\text{P0}}}{\text{2}} \end{array}$$

where *D*_1_ is the first year's dividends, δ is the median of the historical dividend payment rate, and (γ−1) is the long-term growth rate of earnings per share. Following Ohlson and Juettner-Narouth (2005), we set this at 5%. If a negative number appears under the radical, the final equity cost may be a missing value. To avoid this, we process the missing values. That is, we let *Re* equal *A*.

### Variable Definitions and Descriptive Statistics

Table 1 presents the definitions of the key indicators, while Table 2 reports each variable's statistics. As shown in Table 2, judging from the median, the average salary of executives has a slight increasing trend. After taking the logarithm of the value, the mean pay is 14.14, the standard deviation is small, and the standard normal distribution is present. After taking the logarithm, the average value of *CEOpay* is 13.11. The salary of the general manager accounts for a high proportion of the top three values. With respect to control variables, the systemic risk, *beta*, of China's listed companies from 2005 to 2018 remained basically the same as the market, with the mean and median at about 1.141. The maximum value is also only 1.933, which is consistent with the trend of the stock market as a whole. The values for *BM*, book-to-market ratio, indicate that the book values and market values of listed companies in China differ greatly, with a minimum value of 0.1301 and a maximum value of 1.113. Judging by the mean and standard deviation, the distribution is consistent with a normal distribution. From 2005 to 2018, the turnover of listed companies' stocks (*turnover*) is high and the change is large. The average turnover rate is 5.204, the smallest value is only 0.4582, and the maximum value is as high as 19.35, which is consistent with previous research. In terms of scale, China's listed companies are relatively large, with an average *lnsize* of 22.07, but the return on total assets, *roa*, is not high—only 5.45%—and the highest value is 21.25%. The distribution of operating income is extremely uneven, and the level of debt is high. The average value of *lev* reaches 43.51%, the maximum value is close to 1, which is 85.12%, and the minimum value is only 5.43%. There is a large gap in debt levels. Moreover, 22.6% of listed companies are a combination of a chairman and general manager.

**Table 1:** Variable definitions.

**Variables**	**Description**
**Dependent variables**	
*Gorden*	Equity costs, calculated by Gorden model
*PEG*	Equity costs, calculated by PEG model
*OJ*	Equity costs, calculated by OJ model
*CAPM1*	Equity costs, calculated by CAPM model
**Independent variables**	
*Pay*	Executive compensation, nature logarithm of the top three executives' total compensation
*CEOpay*	CEO's compensation, natural logarithm of CEO's personal annual salary
*MPAY2*	Natural logarithm of the median executive compensation in the same industry in the same year
**Moderator variables**	
*Soe*	The final type of controller, one if a firm is state-owned, zero otherwise
**Control variables**	
*Con*	Ratio of executive shareholdings to total shares
*Roa*	Natural logarithm of corporate profitability, measured as net profit with a lag of one period to the average total assets
*Beta*	Stock market volatility β
*Lev*	Natural logarithm of the ratio of long-term debt to total assets
*Insize*	Natural logarithm of total assets at the end of the year.
*BM*	Ratio of book value to market value
*Turnover*	Ratio of the number of yearly shares traded to total shares outstanding
*CBD*	Dummy variable: one if CEO and Chairman of the board are the same person, zero otherwise.
*Age*	Firm age

**Table 2:** Summary statistics.

**Variables**	***N***	**Mean**	**SD**	**Min**	**Median**	**Max**
Gorden	22,484	0.0860	0.0689	0.0034	0.0692	0.3726
PEG	6,833	0.1186	0.0550	0.0269	0.1085	0.3306
CAPM1	19,577	0.1421	0.5177	0.0006	0.1121	1.5420
OJ	6,985	0.1414	0.0591	0.0291	0.1324	0.3649
pay	22,454	14.1400	0.7787	12.1000	14.1600	16.1600
CEOpay	21,595	13.1100	0.8575	5.5540	13.1400	17.3100
beta	19,577	1.1410	0.2595	0.4791	1.1410	1.9330
BM	22,039	0.6294	0.2415	0.1301	0.6328	1.1130
turnover	22,504	5.2040	3.8470	0.4582	4.1680	19.3500
lnsize	22,505	22.0700	1.2640	19.7400	21.9000	26.0500
lev	22,505	0.4351	0.1985	0.0543	0.4362	0.8512
roa	22,503	0.0545	0.0425	0.0018	0.0444	0.2125
CBD	22,505	0.2261	0.4183	0.0000	0.0000	1.0000

*Variables are as defined in Table 1*.

We also conducted the Variance Inflation Factor (VIF) test to test for multicollinearity among the variables. Generally, if the maximum VIF value does not exceed 10, there is no obvious multicollinearity. Our results show that the largest VIF value among the main explanatory variables is far <3, and the VIF value for executive compensation is 1.82. This indicates that there is no serious multicollinearity (results omitted for brevity).

### Equity Cost Trends From 2005 to 2018

**Figure 1:**
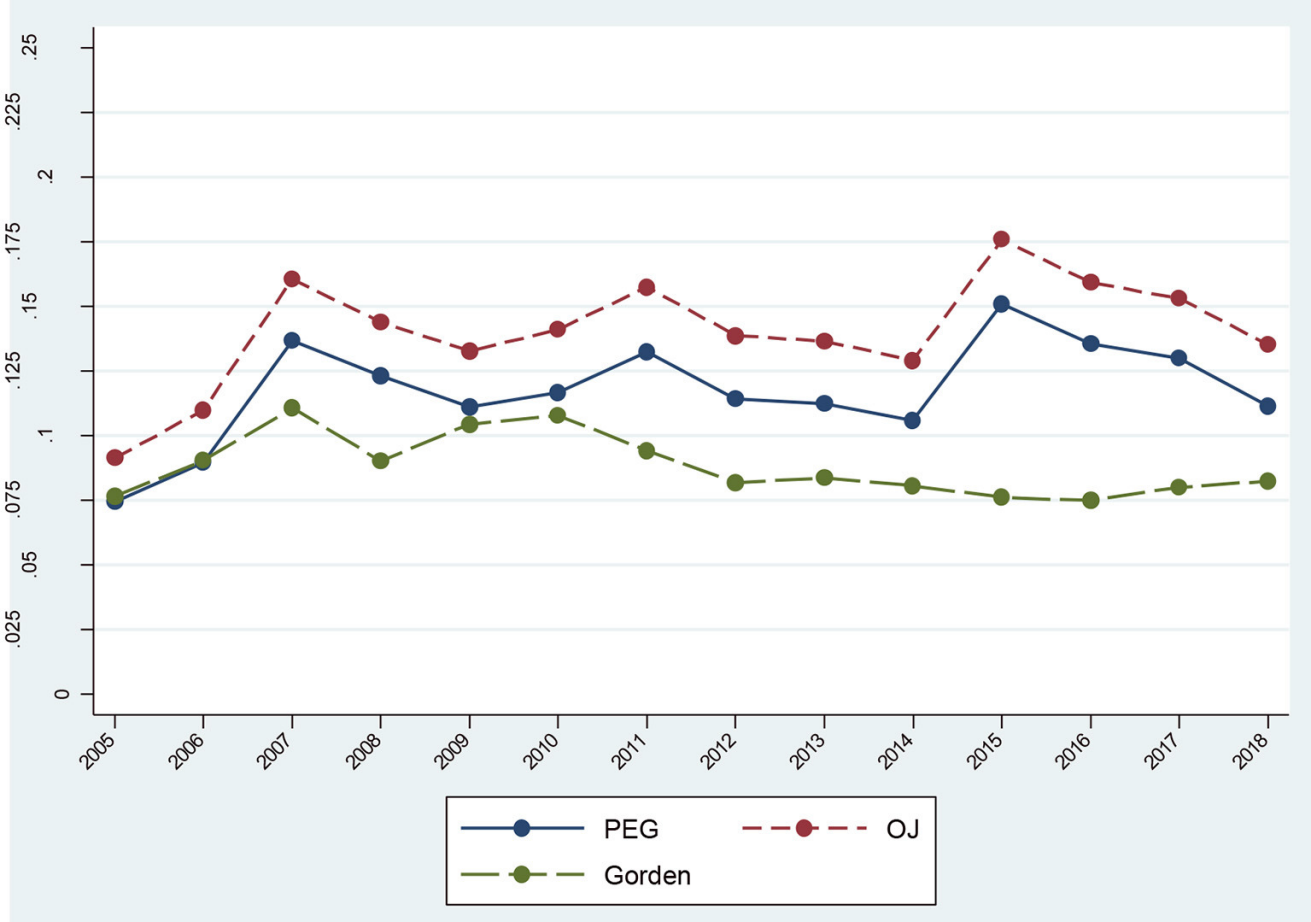
Change trend of equity cost from 2005 to 2018.

Figure 1 shows the trend for equity costs of Chinese listed companies from 2005 to 2018. The results illustrate an “M” type, indicating a significant change in equity cost during this period. Equity cost grew after 2005, reaching a record high in 2007. Upon encountering a financial crisis in 2008, it decreased to its minimum in 2009. Since 2010, it has grown rapidly, reaching a new peak in 2015. After falling slowly, the trend presented a certain change cycle and form, in line with capital market development trends. Figure 1 also indicates that the equity cost of listed companies in China, as a whole, were regular and consistent. When the economy was good and the stock market developed well, the rate of return on investment required by shareholders was higher, which was reflected by the higher cost of equity. When the overall environment was bad, especially in a bear market, the compensation demanded by shareholders was relatively low. Meanwhile, the administrative regulations of the state also played a large role. When the equity cost increased to a certain height, the state would use its “invisible hand” to regulate the market.

**Figure 2:**
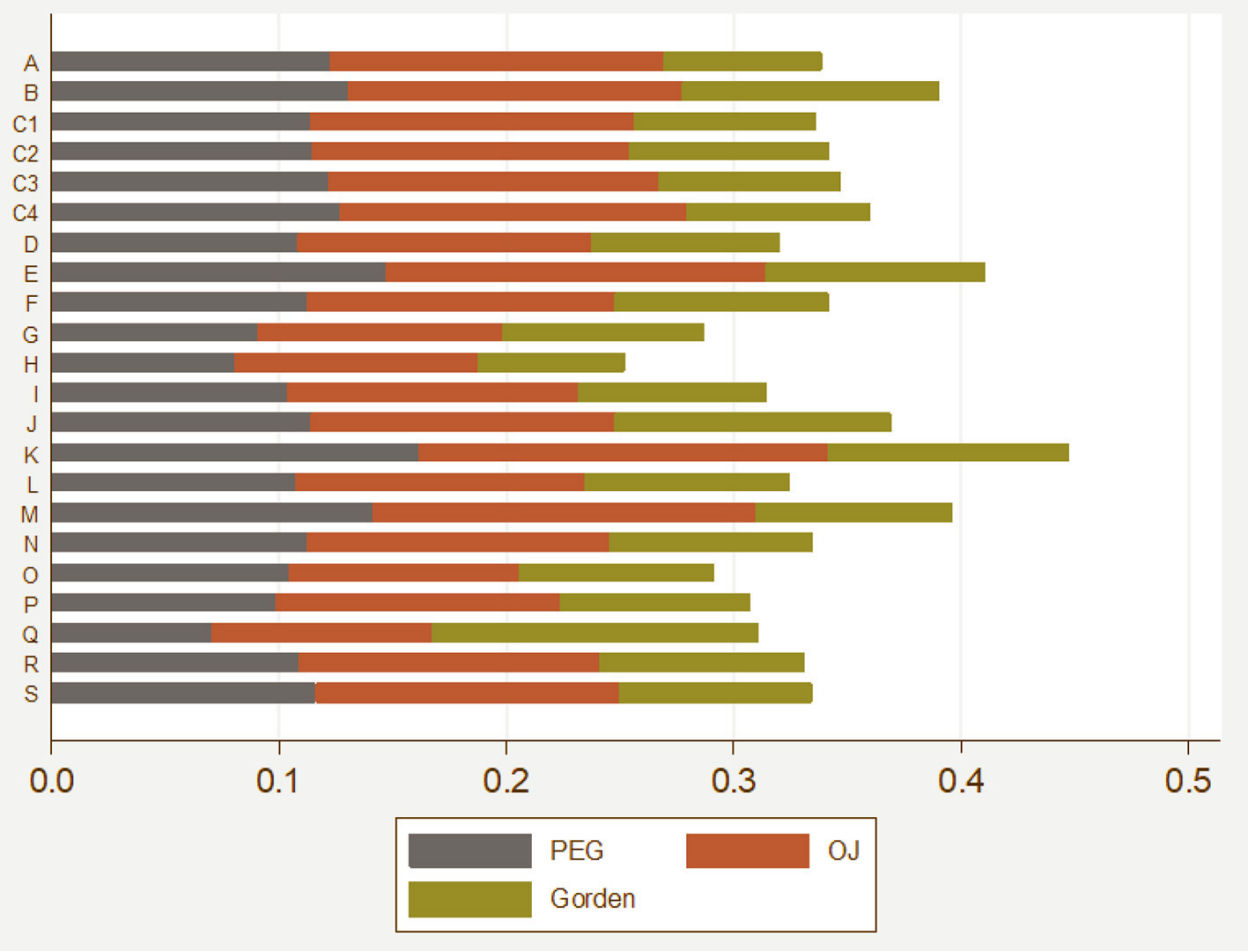
The mean value of equity cost by industry from 2005 to 2018.

Table 3, Figure 2 show comparative results for the mean value of equity cost by industry. The findings indicate that the highest equity cost among Chinese companies was found for Industry K (the real estate industry) from 2005 to 2018. In Figure 2, the average value of equity cost for industry K was the highest, followed by industries B (mining), E (construction), and J (finance). The overall salary for Industry J (the financial industry) was relatively high, but the cost of equity in this industry was not as high as that of the real estate industry. This is in line with China's development; that is, firms in the real estate industry in China suffered severe financing constraints and high financial costs. In addition, Industry H (accommodations, catering) and Industry O (service repair) had the lowest equity costs.

**Table 3:** The mean value of equity cost by industry from 2005 to 2018.

**Industry code**	**Industry name**	**PEG model**		**OJ model**		**Gorden model**	
		***N***	**Mean**	***N***	**Mean**	***N***	**Mean**
A	Farming, forestry, animal husbandry and fishery	102	0.1227	102	0.1468	308	0.0690
B	The mining industry	219	0.1304	232	0.1474	527	0.1129
C1	Food, textile, and leather manufacturing	514	0.1142	520	0.1417	1,594	0.0804
C2	Furniture, papermaking, cultural, and educational petroleum chemistry	1,269	0.1151	1,294	0.1388	4,208	0.0876
C3	Metals, automobiles, railways, computers	2,414	0.1218	2,467	0.1452	7,693	0.0801
C4	Instrumentation and other manufacturing industries	111	0.1271	112	0.1524	447	0.0806
D	Production and supply of electricity, heat, gas, and water	203	0.1084	210	0.1292	841	0.0830
E	The construction industry	200	0.1471	203	0.1672	629	0.0963
F	Wholesale and Retail	377	0.1124	387	0.1353	1,415	0.0936
G	Transportation, warehousing and postal services	254	0.0906	271	0.1079	889	0.0883
H	Accommodation and catering	34	0.0808	35	0.1068	90	0.0644
I	Information transmission and software technology services	463	0.1037	466	0.1279	1,243	0.0824
J	The financial sector	210	0.1142	215	0.1337	495	0.1210
K	The real estate industry	278	0.1612	284	0.1802	1,215	0.1059
L	Leasing and business services	99	0.1070	101	0.1273	276	0.0903
M	Scientific research and technical services	36	0.1415	36	0.1683	141	0.0862
N	Management of water conservancy and environmental public facilities	88	0.1126	90	0.1328	229	0.0896
O	Residents to provide services and repair other service industries	3	0.1044	4	0.1011	39	0.0855
P	Education and its service industries	3	0.0991	3	0.1246	7	0.0835
Q	Health and social work	24	0.0703	24	0.0973	44	0.1426
R	Culture, sports, and entertainment	92	0.1088	92	0.1321	259	0.0902
S	Composite industry	50	0.1163	52	0.1332	390	0.0849
Total	21 categories	7,043	0.1184	7,200	0.1412	22,979	0.0867

As shown in Figure 2, the average value of the cost of equity in all industries in China from 2005 to 2018 was no <10%, and it fluctuated between 10 and 15%. Moreover, the difference in the cost of equity of all industries exhibited little change and certain stability. The results indicate that the cost of equity generally accounted for a large proportion and had regularity. Therefore, the cost of equity should be considered an important factor when making investment decisions.

### Univariate *t*-Test Analysis

Table 4 shows the results of the *t*-tests for executive compensation and equity cost. We classified the full sample into two groups according to property rights. Panel A shows that, due to the implementation of the “salary-restriction order,” executive compensation among private enterprises is significantly higher than that for SOEs in recent years, which is also in line with China's current development situation. In Panel B, firm performance and governance efficiency of SOEs are far lower than that of private enterprises, so the equity cost of private enterprises is significantly lower. This indicates that the innovation of compensation incentive systems in SOEs needs to be continuously promoted.

**Table 4:** Univariate *t*-test.

**Variable**	**Property rights**	**Number**	**Mean**	**Difference**	***t*-value**
Panel A	soe = 0	12,416	14.1644	0.0545	5.2999^***^
Pay	soe = 1	10,038	14.1090		
Panel B	soe = 0	12,420	0.0838	−0.0048	−5.1854^***^
Gorden	soe = 1	10,064	0.0886		

*Variables are as defined in Table 1.*

***, **, and * *represent significance at the l, 5 and 10%, levels, respectively*.

## Empirical Result

### Executive Incentive System and Equity Cost

To test Hypothesis 1 (the impact of executive incentive systems on the cost of equity), following existing research (Core et al., 2008; Hoi et al., 2019; Chu et al., 2020; Pfister et al., 2020; Husser and Paulet, 2021), we used the Gorden and PEG models to measure the equity cost:

(5) $$\begin{array}{l} \overset{4}{\underset{i=1}{\sum }}EC_{it}\;=\;\beta_{0}+\beta_{1}pay_{it-1}+\beta_{2}\ln\;size_{it}+\beta_{3}beta_{it}+\beta_{4}lev_{it}\\ +\;\beta_{5}roa_{it}+\beta_{6}BM_{it}+\beta_{7}CBD_{it}+\beta_{8}turnover_{it}\\ +\;\beta_{9}soe_{it}+\beta_{10}\overset{14}{\underset{i=1}{\sum }}year_{it}+\beta_{11}\overset{21}{\underset{i=1}{\sum }}{industry}_{it} \end{array}$$

where *pay*_*it*−1_ refers to executive compensation with a one-period lag, and EC refers to the cost of equity calculated by the Gorden and PEG models. According to early research, we propose the following control variables: *beta*, the volatility of the stock market; *lnsize*, company size–asset size; *lev*, financial leverage (Eisfeldt and Papanikolaou, 2013); *BM*, book-to-market ratio; *roa*, total return on assets; *turnover*, company stock liquidity; and *CBD*, whether or not the company's two positions are integrated.

Table 5 shows that, after controlling for other factors, all coefficients on *pay* are significant and negative in columns 1 and 2, fully supporting Hypothesis 1. This indicates that higher executive compensation reduces a company's equity cost to a certain extent. According to the principal–agent theory, higher executive compensation tends to mitigate the contradiction between executives and shareholders; executives are likely to work hard for their higher rewards. The cost of equity can be regarded as a reflection of executive ability; specifically, if the level of the company's executive management is higher, firm risks tend to be smaller, and shareholders may require a lower return rate when evaluating investments. As a result, the company tends to pay a lower cost of equity, have more allocated funds to reinvest, create higher value addition, and thereby pay higher compensation to executives.

**Table 5:** Executive incentive and the equity cost.

	**(1)**	**(2)**
**Regressor**	**Gorden model**	**PEG model**
Pay	−0.0019^**^	−0.0060^***^
	(−2.3988)	(−3.9087)
Beta	0.0066^***^	0.0144^***^
	(3.8488)	(4.0666)
Lnsize	0.0006	0.0018
	(0.9514)	(1.4990)
Lev	0.1714^***^	0.0667^***^
	(39.6453)	(9.6525)
Roa	1.5605^***^	0.1888^***^
	(71.3481)	(7.0761)
BM	0.0033	0.0134^**^
	(1.1890)	(2.3123)
Turnover	0.0003^***^	0.0005
	(2.6852)	(1.2521)
CBD	−0.0002	−0.0005
	(−0.1369)	(−0.1857)
Soe	0.0016	−0.0151^***^
	(1.4620)	(−7.1887)
Constant	−0.0732^***^	0.1119^***^
	(−5.1687)	(4.2167)
Year	yes	yes
Industry	yes	yes
Observations	15,492	4,827
R-squared	0.7620	0.2091
Adj. R-squared	0.7614	0.2023

*All variables include: pay, executive compensation taking the 1 period lag; EC, refers to the cost of equity calculated by Gorden model and PEG model; beta, the volatility of the stock market; lnsize, company size–asset size; lev, financial leverage; BM, book to market ratio; roa, total return on assets; turnover, company stock liquidity; CBD, whether the company's two positions are integrated.*

*Z/T statistics are based on a robust corporate clustering standard error.*

***, **, and * *represent significance at the l, 5, and 10%, levels, respectively*.

It can also be seen in Table 5 that, among other control variables, the coefficient between systematic risk (*beta*) and the cost of equity is positive and significant, indicating that the higher the risk of the stock system, the higher the return rate of the stock will be, which is also consistent with the literature. Next, leverage (*lev*) is significantly and positively correlated with equity cost in both models; Fama and French (1993) and some Chinese scholars showed that corporate leverage is directly proportional to stock investment return. In addition, the coefficients on *roa* and *turnover* are significant and positive, indicating that China's current securities market still exhibits the phenomenon of a “small fry”; in other words, many large SOEs and state shares are not valued by the market, and are underestimated.

As we know, entrepreneurs with high professional identity tend to show better innovation performance and higher returns on assets (He et al., 2019; Howell, 2019). Our results prove that the innovation of executive incentive systems is conducive to the creation of a favorable environment for institutional innovation, which helps give full play to entrepreneurship and, thus, reduce corporate costs. The findings are in line with earlier literature (Bosma et al., 2018; Wu and Hsu, 2018; He et al., 2019; Howell, 2019).

### The Moderating Effect of Property Rights

Since the 1980s, China's economic transformation has greatly promoted the vitality of entrepreneurship and private enterprises. Against the background of China's special property rights system, enterprise system innovation not only enhances the competitiveness of enterprises, but also serves as the driving force of a country's sustainable economic growth. China's SOEs, especially the central SOEs, should be the major drivers of social innovation due to their strong financial capacity and policy support. However, the amount of R&D investment and number of patent applications among SOEs are significantly lower than those of non-SOEs, indicating that China has not yet established an effective innovation incentive mechanism in SOEs. As financial decision makers, senior executives have a major influence on corporate financial activities. Therefore, it is necessary to discuss the specific effects of property rights on executive incentive systems and the cost of equity, which can help leaders design an innovative executive incentive system among SOEs.

To further test Hypothesis 2 (the moderating effect of property rights on the relationship between executive incentives and equity cost), we offer the following model:

(6) $$\begin{array}{l} \overset{4}{\underset{i=1}{\sum }}EC_{it}\;=\;\beta_{0}+\beta_{1}pay_{it-1}+\beta_{2}soe+\beta_{3}pay_{it-1}*soe+\beta_{4}beta_{it}\\ +\;\beta_{5}\ln\;size_{it}+\beta_{6}lev_{it}+\beta_{7}roa_{it}+\beta_{8}BM_{it}+\beta_{9}CBD_{it}\\ +\;\beta_{10}turnover_{it}+\beta_{11}\overset{14}{\underset{i=1}{\sum }}year_{it}+\beta_{12}\overset{21}{\underset{i=1}{\sum }}{industry}_{it} \end{array}$$

In columns 1 and 4 of Table 6, we first added the index of property rights, *soe*, and the interaction term between executive pay and property rights, pay^*^soe, to the regressions. As we can see, all coefficients of *pay* are significant and negative at the 1% level, indicating that executive incentives still reduce corporate equity cost after controlling for the effect of property rights. This supports our basic conclusion. Also, pay^*^soe is significantly and positively correlated with equity cost in both models. A one-standard-deviation increase in pay^*^soe increases equity cost by 0.0076 in Column 1, whereas it increases equity cost by 0.0041 in Column 4. The results suggest that property rights tend to weaken the negative effect of executive incentives on equity cost, supporting Hypothesis 2.

**Table 6:** The moderating effect of property rights.

**Regressor**	**(1)** **Gorden model**			**(2)** **PEG model**		
	**Full sample**	**soe = 1**	**soe = 0**	**Full sample**	**soe = 1**	**soe = 0**
pay	−0.0055^***^	0.0019	−0.0054^***^	−0.0079^***^	−0.0002	−0.0114^***^
	(−5.8888)	(1.6292)	(−5.3344)	(−4.3091)	(−0.1014)	(−5.2315)
soe	−0.1061^***^			−0.0734^**^		
	(−6.2414)			(−2.4177)		
pay*soe	0.0076^***^			0.0041^*^		
	(6.2562)			(1.9292)		
beta	0.0065^***^	0.0030	0.0096^***^	0.0143^***^	0.0221^***^	0.0029
	(3.8153)	(1.1641)	(4.2345)	(4.0492)	(4.6093)	(0.5890)
lnsize	0.0005	−0.0018^**^	0.0026^**^	0.0017	0.0005	0.0043^**^
	(0.7133)	(−2.1090)	(2.2508)	(1.4235)	(0.3374)	(2.0729)
lev	0.1723^***^	0.1817^***^	0.1625^***^	0.0677^***^	0.0751^***^	0.0541^***^
	(40.0523)	(28.2668)	(28.3497)	(9.7941)	(8.0907)	(5.0369)
roa	1.5606^***^	1.7111^***^	1.4420^***^	0.1894^***^	0.2573^***^	0.1337^***^
	(71.7548)	(52.5388)	(50.7502)	(7.1045)	(7.0099)	(3.5670)
BM	0.0031	0.0145^***^	−0.0044	0.0130^**^	0.0174^**^	0.0123
	(1.1120)	(3.7604)	(−1.0835)	(2.2551)	(2.1334)	(1.4419)
turnover	0.0003^***^	0.0003	0.0004^**^	0.0005	0.0008	0.0001
	(2.6539)	(1.3230)	(2.5011)	(1.2373)	(1.4481)	(0.2452)
CBD	0.0001	−0.0010	0.0011	−0.0003	−0.0010	−0.0008
	(0.0999)	(−0.5547)	(0.8082)	(−0.1280)	(−0.2289)	(−0.2688)
Constant	−0.0181	−0.0846^***^	−0.0533^**^	0.1421^***^	0.0376	0.1476^***^
	(−1.1094)	(−4.2470)	(−2.2787)	(4.6000)	(1.0665)	(3.6215)
Year	Yes	Yes	Yes	Yes	Yes	Yes
Industry	Yes	Yes	Yes	Yes	Yes	Yes
Observations	15,492	8,103	7,389	4,827	2,478	2,349
R-squared	0.7638	0.7856	0.7525	0.2100	0.2274	0.2119
Adj. R-squared	0.7632	0.7846	0.7512	0.2031	0.2153	0.1986

*All variables include: pay, executive compensation taking the 1 period lag; EC, refers to the cost of equity calculated by Gorden model and PEG model; beta, the volatility of the stock market; lnsize, company size–asset size; lev, financial leverage; BM, book to market ratio; roa, total return on assets; turnover, company stock liquidity; CBD, whether the company's two positions are integrated.*

*Z/T statistics are based on a robust corporate clustering standard error.*

***, **, and * *represent significance at the l, 5, and 10%, levels, respectively*.

Furthermore, we classified the full sample into SOEs and private companies according to the final controllers, *soe*. Columns 2, 3, 5, and 6 of Table 6 show the comparison results between two sub-samples. The coefficients of executive compensation (*pay*) are significant and negative at the 1% level for the private-enterprise group, but negative and non-significant for the SOE group. This indicates that the effect of executive incentives on equity cost in private enterprises is more obvious. It may be explained by the special nature of China's SOEs, executives' direct appointment by the government and compensation systems that are more regulated by the government. However, private enterprises are not constrained by political tasks, and their development is more in line with the practical logic of the market and corporate governance. Although the development of China's current manager market is relatively slow, the role of the manager market is being reflected in private enterprises in recent years, and the efficiency of listed private enterprises is often higher than that of the SOEs.

### Endogeneity Problem

To eliminate the endogeneity problem caused by omitted variables, this study used the two-stage least squares method (2SLS). We regard the median of executive salary in the same industry in the same year, *MPAY2*, as an instrumental variable of executive compensation to conduct the 2SLS regression. The results are shown in Table 7.

**Table 7:** Endogeneity problem: 2SLS.

**Regressor**	**Gorden model**		**PEG model**	
	**First stage**	**Second stage**	**First stage**	**Second stage**
	**(1)**	**(2)**	**(3)**	**(4)**
MPAY2	0.5462^***^		0.5621^***^	
	(22.6430)		(12.6704)	
pay		−0.0096^***^		−0.0177^***^
		(−3.6600)		(−2.7762)
beta	0.1181^***^	0.0074^***^	0.0705^*^	0.0150^***^
	(5.3621)	(5.5183)	(1.8012)	(4.7222)
lnsize	0.2704^***^	0.0029^***^	0.2491^***^	0.0049^**^
	(43.5693)	(3.4091)	(21.7435)	(2.5604)
lev	−0.1015^***^	0.1705^***^	0.0280	0.0667^***^
	(−2.9925)	(83.3993)	(0.4140)	(12.2016)
roa	2.5845^***^	1.5811^***^	2.4491^***^	0.2181^***^
	(18.6543)	(146.0873)	(9.6367)	(8.3953)
BM	−0.3392^***^	0.0001	−0.2389^***^	0.0096^*^
	(−10.6874)	(0.0602)	(−3.9675)	(1.8310)
turnover	−0.0152^***^	0.0002^**^	−0.0169^***^	0.0003
	(−8.4831)	(2.0049)	(−4.3443)	(0.8199)
CBD	0.0522^***^	0.0003	0.0748^***^	0.0004
	(4.1942)	(0.3913)	(3.3202)	(0.2347)
soe	0.0147	0.0017^***^	−0.0285	−0.0155^***^
	(1.3839)	(2.6650)	(−1.4123)	(−9.4388)
Constant	0.1970	−0.0160	0.3993	0.1876^***^
	(0.6041)	(−0.8061)	(0.6555)	(3.6379)
Year	Yes	Yes	Yes	Yes
Industry	Yes	Yes	Yes	Yes
Observations	15,492	15,492	4,827	4,827
R-squared	0.4658	0.7578	0.4686	0.1917
Adj. R-squared	0.4644	0.7572	0.4640	0.1848

*All variables include: MPAY2, an instrumental variable of executive compensation; pay, executive compensation taking the 1 period lag; EC, refers to the cost of equity calculated by Gorden model and PEG model; beta, the volatility of the stock market; lnsize, company size–asset size; lev, financial leverage; BM, book to market ratio; roa, total return on assets; turnover, company stock liquidity; CBD, whether the company's two positions are integrated.*

*Z/T statistics are based on a robust corporate clustering standard error.*

***, **, and * *represent significance at the l, 5, and 10%, levels, respectively*.

In Table 7, *MPAY2* is significantly and positively correlated with executive compensation in columns 1 and 3. A one-standard-deviation increase in *MPAY2* increases executive compensation by 0.5462 in Column 1, whereas it increases executive compensation by 0.5621 in Column 3. This indicates that the instrumental variable is highly correlated with executive compensation. The adjusted *R*^2^-values of 0.4644 and 0.4640 indicate that *MPAY2*, as the instrumental variable, has good explanatory power. Furthermore, columns 2 and 4 show the results of the second-stage regression. The coefficients for executive compensation are still significant and negative at the 1% level, indicating that higher executive compensation is likely to reduce the company's equity cost. This indicates that our conclusions are robust.

## Robustness Tests

### Alternative Dependent Variable: CAPM, OJ

To better verify the impact of executive compensation on the cost of equity, we used the CAPM model and the OJ model to remeasure equity cost. As can be seen from Table 8, the coefficients on executive compensation and equity cost are significant and negative at the 1 and 5% levels, respectively. Our conclusion is still valid.

**Table 8:** Alternative dependent variable: CAPM, OJ.

**Regressor**	**(1)** **CAPM model**			**(2)** **OJ model**		
	**Full sample**	**soe = 1**	**soe = 0**	**Full sample**	**soe = 1**	**soe = 0**
pay	−0.0058^***^	−0.0059^***^	−0.0065^***^	−0.0040^**^	0.0019	−0.0094^***^
	(−3.3918)	(−2.6538)	(−2.5798)	(−2.4728)	(0.9182)	(−3.9163)
beta	0.0951^***^	0.1274^***^	0.0629^***^	0.0118^***^	0.0191^***^	0.0014
	(14.2192)	(13.5601)	(6.5753)	(3.0631)	(3.5925)	(0.2687)
lnsize	0.0186^***^	0.0206^***^	0.0162^***^	0.0020	0.0015	0.0030
	(12.9170)	(11.2655)	(6.7874)	(1.4772)	(0.8928)	(1.3055)
lev	−0.0339^***^	−0.0342^***^	−0.0369^***^	0.0583^***^	0.0634^***^	0.0517^***^
	(−4.6232)	(−3.5387)	(−3.1678)	(7.8600)	(6.3081)	(4.4996)
roa	−0.1978^***^	−0.2328^***^	−0.1797^***^	0.1695^***^	0.1995^***^	0.1468^***^
	(−5.9766)	(−4.9468)	(−3.9862)	(5.7627)	(4.6715)	(3.6071)
BM	−0.0371^***^	−0.0403^***^	−0.0308^***^	0.0069	0.0059	0.0108
	(−5.9981)	(−4.4756)	(−3.6170)	(1.1053)	(0.6642)	(1.1624)
turnover	0.0043^***^	0.0054^***^	0.0033^***^	0.0003	0.0004	0.0001
	(9.0980)	(7.4656)	(5.1646)	(0.8291)	(0.7366)	(0.1203)
CBD	−0.0026	−0.0081^*^	−0.0004	−0.0003	−0.0025	0.0002
	(−1.0732)	(−1.7881)	(−0.1375)	(−0.0998)	(−0.5296)	(0.0547)
soe	−0.0014			−0.0162^***^		
	(−0.6365)			(−7.1825)		
Constant	0.5757^***^	−0.6745^***^	0.6668^***^	0.1049^***^	0.0319	0.1800^***^
	(17.9466)	(−14.9123)	(12.9584)	(3.7177)	(0.8461)	(4.0873)
Year	Yes	Yes	Yes	Yes	Yes	Yes
Industry	Yes	Yes	Yes	Yes	Yes	Yes
Observations	15,495	8,103	7,392	4,924	2,546	2,378
R-squared	0.9701	0.9738	0.9645	0.1763	0.1833	0.1792
Adj. R-squared	0.9700	0.9737	0.9643	0.1693	0.1709	0.1655

*All variables include: pay, executive compensation taking the 1 period lag; EC, refers to the cost of equity calculated by Gorden model and PEG model; beta, the volatility of the stock market; lnsize, company size-asset size; lev, financial leverage; BM, book to market ratio; roa, total return on assets; turnover, company stock liquidity; CBD, whether the company's two positions are integrated.*

*Z/T statistics are based on a robust corporate clustering standard error.*

***, **, and * *represent significance at the l, 5, and 10%, levels, respectively*.

### Alternative Independent Variable: CEOpay

The general manager (CEO) plays an important role in the senior management team of Chinese listed companies. CEOs are regarded as valuable organizational human capital. Efficient corporate human capital can bring great benefits to the company. Therefore, we take the logarithm of the personal salary of the general manager and CEO, *CEOpay*, as an alternative to executive compensation *pay*. Table 9 shows that the coefficients on *CEOpay* are significant and negative at the 5 and 1% levels, respectively. This suggests that the negative effect of the CEO's compensation incentive system on equity cost is still significant, and more so in private enterprises. Our basic conclusions remain.

**Table 9:** Alternative independent variable: CEOpay.

**Regressor**	**(1)** **Gorden model**			**(2)** **PEG model**		
	**Full sample**	**soe = 1**	**soe = 0**	**Full sample**	**soe = 1**	**soe = 0**
CEOpay	−0.0015^**^	0.0011	−0.0039^***^	−0.0046^***^	0.0008	−0.0096^***^
	(−2.2459)	(1.0951)	(−4.4696)	(−3.4116)	(0.4576)	(−4.8646)
beta	0.0063^***^	0.0026	0.0096^***^	0.0152^***^	0.0241^***^	0.0030
	(3.5945)	(0.9789)	(4.1463)	(4.1398)	(4.8406)	(0.5765)
lnsize	0.0007	−0.0013	0.0021^*^	0.0009	−0.0006	0.0035^*^
	(0.9743)	(−1.5290)	(1.8164)	(0.7528)	(−0.3757)	(1.7196)
lev	0.1707^***^	0.1810^***^	0.1619^***^	0.0652^***^	0.0725^***^	0.0522^***^
	(38.9907)	(27.7582)	(27.5802)	(9.2135)	(7.5888)	(4.7889)
roa	1.5508^***^	1.7062^***^	1.4332^***^	0.1901^***^	0.2638^***^	0.1328^***^
	(69.8653)	(51.3913)	(49.9301)	(7.0101)	(7.0249)	(3.5123)
BM	0.0029	0.0130^***^	−0.0037	0.0146^**^	0.0200^**^	0.0140
	(1.0406)	(3.3075)	(−0.8993)	(2.4978)	(2.4508)	(1.6212)
turnover	0.0004^***^	0.0003	0.0004^***^	0.0004	0.0006	0.0002
	(2.6981)	(1.2641)	(2.6026)	(1.1117)	(1.0983)	(0.3267)
CBD	−0.0001	−0.0006	0.0009	−0.0000	−0.0010	−0.0003
	(−0.0956)	(−0.3073)	(0.6453)	(−0.0179)	(−0.2212)	(−0.1017)
soe	0.0017			−0.0146^***^		
	(1.5700)			(−6.9650)		
Constant	−0.0796^***^	−0.0772^***^	−0.0642^***^	0.0955^***^	0.0472	0.1186^***^
	(−5.6084)	(−3.9131)	(−2.7279)	(3.6735)	(1.3669)	(2.9749)
Year	Yes	Yes	Yes	Yes	Yes	Yes
Industry	Yes	Yes	Yes	Yes	Yes	Yes
Observations	14,834	7,640	7,194	4,624	2,322	2,302
R-squared	0.7606	0.7840	0.7513	0.210	0.236	0.211
Adj. R-squared	0.7599	0.7829	0.7499	0.203	0.223	0.197

*All variables include: CEOpay, CEO's compensation; EC, refers to the cost of equity calculated by Gorden model and PEG model; beta, the volatility of the stock market; lnsize, company size–asset size; lev, financial leverage; BM, book to market ratio; roa, total return on assets; turnover, company stock liquidity; CBD, whether the company's two positions are integrated.*

*Z/T statistics are based on a robust corporate clustering standard error.*

***, **, and * *represent significance at the l, 5, and 10%, levels, respectively*.

### Considering the Effect of Executive Shareholdings

Considering the effect of executive shareholdings, we added the index of executive shareholdings, *con*, to the regression. The results in Table 10 show that the negative effect of executive compensation is still significant after controlling for the effect of executive shareholdings, further supporting our basic conclusion.

**Table 10:** Considering the effect of executive shareholdings.

**Regressor**	**(1)** **Gorden model**			**(2)** **PEG model**		
	**Full sample**	**soe = 1**	**soe = 0**	**Full sample**	**soe = 1**	**soe = 0**
pay	−0.0018^**^	0.0021^*^	−0.0052^***^	−0.0059^***^	0.0000	−0.0115^***^
	(−2.2780)	(1.7487)	(−5.1253)	(−3.7496)	(0.0227)	(−5.1904)
con	−0.0172^***^	−0.1586^***^	−0.0118^**^	0.0245^**^	0.0408	0.0285^**^
	(−3.5225)	(−2.9015)	(−2.3691)	(2.2599)	(0.7898)	(2.5387)
beta	0.0065^***^	0.0033	0.0091^***^	0.0144^***^	0.0225^***^	0.0032
	(3.6936)	(1.1936)	(4.0298)	(3.9589)	(4.4290)	(0.6442)
lnsize	0.0006	−0.0019^**^	0.0025^**^	0.0022^*^	0.0008	0.0048^**^
	(0.8468)	(−2.1655)	(2.1077)	(1.7156)	(0.4908)	(2.2862)
lev	0.1709^***^	0.1832^***^	0.1614^***^	0.0680^***^	0.0741^***^	0.0580^***^
	(38.4905)	(27.3764)	(27.9184)	(9.7529)	(7.5786)	(5.6395)
roa	1.5576^***^	1.7191^***^	1.4394^***^	0.1894^***^	0.2559^***^	0.1392^***^
	(68.7708)	(49.8400)	(49.6778)	(6.9768)	(6.6320)	(3.7277)
BM	0.0037	0.0142^***^	−0.0039	0.0115^*^	0.0163^*^	0.0100
	(1.2975)	(3.5809)	(−0.9380)	(1.9413)	(1.9361)	(1.1621)
turnover	0.0004^***^	0.0002	0.0005^***^	0.0005	0.0008	0.0001
	(2.8980)	(1.1685)	(2.7125)	(1.1520)	(1.4590)	(0.1065)
CBD	0.0011	−0.0006	0.0022	−0.0022	−0.0016	−0.0035
	(0.8826)	(−0.3117)	(1.4217)	(−0.8465)	(−0.3620)	(−1.0868)
soe	0.0011			−0.0139^***^		
	(0.9805)			(−6.3965)		
Constant	−0.0771^***^	−0.0787^***^	−0.0516^**^	0.0902^***^	−0.0021	0.1207^***^
	(−5.2332)	(−3.9330)	(−2.1790)	(3.3889)	(−0.0558)	(2.9857)
Year	Yes	Yes	Yes	Yes	Yes	Yes
Industry	Yes	Yes	Yes	Yes	Yes	Yes
Observations	14,729	7,565	7,164	4,612	2,319	2,293
R-squared	0.7593	0.7812	0.7532	0.2090	0.2238	0.2159
Adj. R-squared	0.7586	0.7800	0.7518	0.2017	0.2105	0.2019

*All variables include: pay, executive compensation taking the 1 period lag; EC, refers to the cost of equity calculated by Gorden model and PEG model; beta, the volatility of the stock market; lnsize, company size-asset size; lev, financial leverage; BM, book to market ratio; roa, total return on assets; turnover, company stock liquidity; CBD, whether the company's two positions are integrated.*

*Z/T statistics are based on a robust corporate clustering standard error.*

***, **, and * *represent significance at the l, 5, and 10%, levels, respectively*.

### Sample Selection

To eliminate the impact of the new accounting standards in 2007, we reselected data from 2008 to 2018 as the sample period and re-ran the regression. The conclusions are unchanged.

### Alternative Dependent Variable: Historically Annual Closing Price

We substituted the historically annual closing price for the latest closing price to measure equity cost in the OJ and PEG models and re-ran the models. Our results are still robust.

## Discussion

Our research empirically supports hypotheses 1 and 2. The results confirm that higher executive compensation reduces a company's equity cost to a certain extent, thus supporting Hypothesis 1. Higher executive compensation tends to mitigate the contradiction between executives and shareholders. Moreover, Hypothesis 2 is also supported through our observation of the more significant effect of executive incentives on equity cost in private enterprises. Private enterprises are not constrained by political tasks, and their development is more in line with the practical logic of the market.

Entrepreneurship is the inner impulse of entrepreneurs to pursue innovation, and it has a significant impact on corporate behavior and financial decisions, promoting the differentiation of enterprises. The hard work by enterprise executives, especially founders, in the process of enterprise establishment and development make them less likely to engage in negative self-interested behaviors, such as slacking off, thus alleviating the agency problem. Entrepreneurs with high professional identity tend to show better innovation performance and return on assets. Our research proves that the innovation of the executive incentive system is conducive to the creation of a favorable environment for institutional innovation, which helps give full play to entrepreneurship, thus reducing corporate costs.

Executive incentive system innovation can more effectively stimulate the entrepreneurial spirit and improve the level of corporate innovation. The amount of R&D investment and the number of patent applications among non-SOEs are significantly higher than those of SOEs, indicating that China has not yet established an effective innovation incentive mechanism for SOEs. As the final decision makers on various financial policies, senior executives have a decisive influence on corporate financial activities. Therefore, it is of great practical significance to conduct a research an innovation-oriented incentive system for senior executives among Chinese enterprises, especially SOEs.

## Conclusion

Using 15,492 firm-year observations from China's Shanghai and Shenzhen A-share listed companies for the period between 2005 and 2018, we constructed various models, including the Gorden model, the PEG model, the CAPM model, and the OJ model, to measure corporate equity cost. We investigated the effects of executive compensation incentives on the cost of equity, and further explore the effects of China's special property rights system on this relationship.

This study is particularly significant because it proves that executive incentive systems and corporate governance mechanisms in China's SOEs are neither complete nor efficient. It is necessary to introduce more innovative competition factors in the manager market, give full play to the role of executive compensation incentives, and integrate the executive incentives and capital cost of the company. When these are closely linked, research can better reflect the most basic economic significance of a company's financial management, actively promoting the innovation of executive incentives systems in SOEs. Consequently, the operating efficiency of state-owned assets, such as SOEs and large enterprises, will be improved.

In corporate governance practice, achieving a positive interaction between the investor protection and the management's abilities is always the main thrust of corporate governance. A company's scientific and efficient executive incentive system must incorporate managers' human capital and investors' interests. Only dynamic and real-time constraints and incentives for corporate executives can be combined organically, creating firm value while maximizing investors' interests. The company must be fully aware of the effects of executive personal characteristics on corporate innovation and other financial decisions. Also, the company should establish an innovative executive incentive mechanism to motivate executives' efficiency, improve firm value, reduce equity cost, and promote corporate innovation. Therefore, the innovation of executive incentive mechanisms is an inevitable choice to promote enterprises' competitiveness and creativity, and it is an important measure to optimize the urban economic structure and achieve high-quality economic growth in China. The interaction between system innovation, enterprise entrepreneurship, and financial behavior should be the ultimate goal of sustainable development and value maximization among enterprises.

## Data Availability Statement

The raw data supporting the conclusions of this article will be made available by the authors, without undue reservation.

## Author Contributions

JW: collected the data and designed the research. JD: analyzed the data and empirical results. JW and JD: wrote the manuscript. All authors contributed to the article and approved the submitted version.

## Conflict of Interest

The authors declare that the research was conducted in the absence of any commercial or financial relationships that could be construed as a potential conflict of interest.
